# Advanced Nano-Carriers for Anti-Tumor Drug Loading

**DOI:** 10.3389/fonc.2021.758143

**Published:** 2021-09-16

**Authors:** Jia Xiang, Rui Zhao, Bo Wang, Xinran Sun, Xu Guo, Songwen Tan, Wenjie Liu

**Affiliations:** Xiangya School of Pharmaceutical Sciences, Central South University, Changsha, China

**Keywords:** cancer, chemotherapy, antitumor drug, nano drug carrier, targeted transportation

## Abstract

Chemotherapy is one of the important means of tumor therapy. However, most of the anti-tumor drugs that currently used in clinic are hydrophobic non-specific drugs, which seriously affect the efficacy of drugs. With the development of nanotechnology, drug efficacy can be improved by selecting appropriate biodegradable nanocarriers for achieving the controlled release, targeting and higher bioavailability of drugs. This paper reviewed the research progress of anti-tumor drug nanoparticle carriers, which mainly summarized the materials used for anti-tumor drug nanoparticle carriers and their effects in anti-tumor drugs, as well as the targeted drug delivery methods of anti-tumor drugs based on nanocarriers.

## Introduction

Tumor is still one of the main fatal diseases of human beings, and chemotherapy is one of the main methods to treat tumor in clinic (1). However, most anti-tumor drugs have poor water solubility and low bioavailability (2), which leads to the limited therapeutic effect of drugs on tumor tissues (3). In addition, chemotherapy will have serious toxic and side effects on other normal tissues and cells (4), which not only damages patients’ body function, but also causes patients to develop drug resistance (4), which seriously affects the curative effect. In 1978, Marty first used nanoparticles as drug carriers (5). Nanocarriers are nano-sized carriers based on the concept of targeted drug delivery system (TDDS) (4). At present, it has become a research hotspot due to its advantages of controlled release, targeting, high efficiency, low toxicity and high stability (6). With the development of nanotechnology, the anti-tumor drug with nanoparticles as carriers can achieve controlled release and targeted drug delivery through using special materials and surface modification (4). It can also improve the stability and bioavailability of anti-tumor drugs, overcome the limitations of traditional anti-tumor drugs, which has been widely used. In this review, we introduced main types of nano-drug carrier materials and their effects and discussed the targeted transport modes of nano-drug carriers.

## Nano Carriers for Anti-Tumor Drug

**Figure 1:**
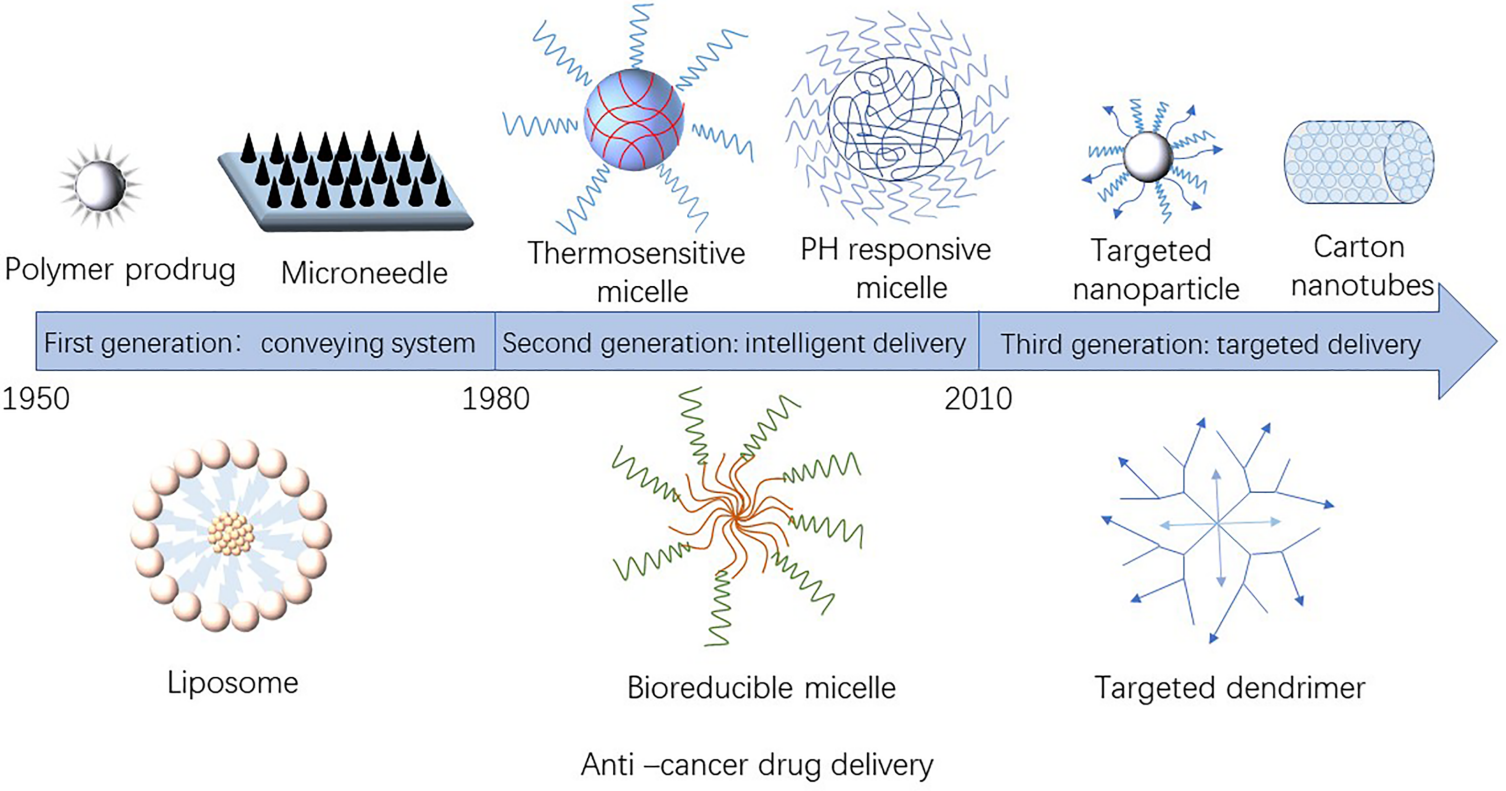
Development of nano-carriers for anti-tumor drug.

The nanoparticles used as anti-tumor drug loading system have a size of 1–100 nm (4) and mainly include nano-liposomes, nano-polymers, nano-gene carriers, nano-inorganic materials and other drug carriers. **Figure 1** showed the development of nano-sized anti-tumor drug carriers. **Table 1** presented the materials used as anti-tumor drugs delivery carriers.

**Table 1:** Different materials as anti-tumor drugs delivery carrier.

Nanomaterial	Modification	Anti-cancer drug	Results	Reference
**Liposomes**	No	Luteolin	Improve solubility and bioavailability of Luteolin and obtain better anticancer effect	(7)
	Anti-GD2 antibodies	Doxorubicin	Improve the targeting of drugs to Human Neuroblastoma	(8)
	NGR peptides	Bortezomib	Reduce side effects and improve anti-tumor effect	(9)
**hyaluronic**	Chitosan	Paclitaxel	Improve the targeting and stability of drug	(10)
**PLA**	No	Tamoxifen	Reduce side effects and improve anti-tumor effect	(11)
	methoxy poly (ethylene glycol)	verapamil and doxorubicin	Reduce drug resistance and improve anti-cancer effect	(12)
	PEG	Anastrozole	Effectively deliver Anastrozole to target cells	(13)
**PLGA**	Chitosan and PEG	curcumin	Improve the targeting of drug	(14)
**PEG**	Fe_3_O_4_	doxorubicin	Improve the therapeutic effect	(15)
**Poly(anhydride)**	Cyclodextrins	Camptothecin	Improve bioavailability of Camptothecin	(16)
**Chitosan**	No	Chlorambucil	Reduce the abnormal toxicity and enhance the uptake of tumor cells	(17)
**carbon nanotubes**	Folate (covalent)	Doxorubicin and cell impermeant propidium iodide	Improve the drug loading and stability	(18)
	Fluorescent probe labeled single strand DNA (non-covalent)	No	Improve the targeting response ability	(19)
**Silica Nanoparticles**	Econazole	Fluconazole	Enhance targeting and permeability and reduce side effects	(20)
**Au nanorods**	5’ thiol end	Doxorubicin and platinum	Improve the targeting and response activity	(21)
**ZnO nanoparticle**	PEG	Doxorubicin	Increase blood concentration and anti-tumor efficiency	(22)
	No	Ibuprofen	Reduce dissolution rate and improve anti-tumor ability	(23)
**Quantum dots**	Boron nitride	Doxorubicin	Improve drug activity and anti-tumor ability	(24)

### Nano-Liposomes

Liposomes are structures, similar to biofilms, composed of hydrophilic head and lipophilic tail, which can encapsulate drugs in aqueous solution to form monolayer or multilayer vesicles (25). Liposomes are considered as promising drug carriers due to their low toxicity, high safety, high biocompatibility, strong drug loading capacity and more flexible regulation of drug release (25–27). Luteolin (LUT) is a kind of natural flavonoids widely distributed in a variety of plants and has anti-tumor activity. Due to the poor water solubility and low bioavailability of luteolin, so Wu et al. (7) prepared liposome LUT with encapsulation efficiency of up to 90%. *In vitro* studies show that LUT encapsulated by liposome can inhibit tumor growth by inducing apoptosis of tumor cells and has superior anti-tumor effect on mouse colon cancer cell CT26 compared with LUT without liposome.

### Nano-Polymers

Nano-polymer carriers have good biocompatibility and biodegradability (28), and have been widely studied in the medical field. Currently, nano-polymer carriers used for anti-tumor drugs mainly divided into natural and synthetic nano-polymer carriers. Natural nano-polymer carriers mainly include hyaluronic acid-based polymers, agarose, collagen and chitosan while synthetic nano-polymer carriers include poly anhydrides, poly (ϵ-caprolactone) (PCL), polylactic acid (PLA), polyethylene glycol (PEG), polyglutamic acid(PGA),and poly D,L-lactide-co-glocolide(PLGA),etc. Among them, PEG with low toxicity have been the focus of research in recent years. PEG is a polymer material obtained by ring-opening polymerization of ethylene oxide. Its main characteristics are controllable polymerization degree, stable structure (29), and can avoid the recognition of human immune system, which has the property of “stealth” *in vivo*. Genexol-PM^®^ in Korea and Paclical^®^ in Russia are polymer nanodrugs that have been approved clinically, and both of them are polymer nanomedicine formulations of paclitaxel. Furthermore, there are many polymer nano-drugs that have entered preclinical research (30), such as Opaxio and Xyotax.

### Nano-Gene Carriers

Gene therapy played an important role in cancer treatment (31). The carrier of gene therapy was the key to the success of gene therapy (32). At present, the commonly used gene vectors mainly include viral vectors and non-viral vectors. The difficulty and high cost in preparing viral vectors and the potential carcinogenicity limit their application in gene therapy (33). As a potential substitute for viral vectors, non-viral vectors are simple to prepare, high in portability and low in toxicity, and most of them are nano-vectors including peptides, liposome, polymers and so on (34–36). Wang et al. (37) prepared a multifunctional tumor therapeutic carrier transport plasmid Cas9-sgPlk-1 by electrostatic interaction with lipid encapsulated gold nanoparticles, which provides a multifunctional method for efficient targeted gene editing and makes nano-gene carriers more widely used *in vivo* and *in vitro* experiments.

### Inorganic Nanoparticles

In recent years, inorganic nanoparticles have been widely used in tumor imaging and treatment (38) mainly including metals (e.g., gold, zinc, silver and iron nanoparticles), metal oxides (iron and titanium oxide nanoparticles), carbon dots, carbon nanotubes and semiconductors etc. (39, 40). Due to the unique physical and chemical properties and good stability of inorganic nanoparticles, especially optical, magnetic and other physical properties, inorganic nanoparticles may be more suitable for cancer treatment than traditional organic nanocarriers (38). Chen et al. (41) modify Fe_3_O_4_ nanoparticles onto carbon nanotubes to provide a double-targeted drug delivery system with about 110% excellent drug-loading capacity for tumor-targeted optical imaging and magnetic-targeted drug delivery. However, the toxicity of inorganic nanoparticles greatly limits its clinical application (42).

## Targeted Delivery of Nano-Drug Carriers

Although traditional chemotherapy drugs can kill tumor cells with high efficiency, they also have toxic and side effects on normal tissues due to their lack of specificity while nanotechnology provides a new opportunity for tumor targeted therapy (43). At present, anti-tumor drugs based on nanoparticles can be targeted to transport drugs through three ways: passive transport, active transport and physical and chemical transport, which can identify cancerous tissues more accurately in complex organisms and release them at cancerous tissues to reduce toxic and side effects on normal cells.

### Passive Targeted Transport

Passive targeting, mainly through permeation and retention effect(EPR), enables the drug to be swallowed by macrophages as a foreign body immediately after entering the human body, so as to reduce non-specific binding with non-target sites and reach the targeted sites for selective binding (44). Drug carriers, such as liposomes, mainly transported drugs through passive targeting (45). Mitra et al. (46) embedded adriamycin glucan complex in long-circulating nanoparticles, and enriched the drug targeting to the tumor site of mice by EPR effect, so as to achieve the purpose of slow targeting, high efficiency and low toxicity of drugs. At present, many passive targeting nanoparticles have shown promising therapeutic effects in clinical trials, such as Marqibo, Myocet and lysosomes (47).

### Active Targeted Transport

The limitation of passive targeting is that it has lower specificity to tumor site, while active targeting has higher targeting. It is found that some antigens or receptors are over-expressed on the surface of tumor cells, while normal cells express them normally (48), such as folate receptor (49), prostate-specific membrane antigen (50), biotin receptors (51), transferrin receptor (52), peptide (53) and the carbonic anhydrase IX (54). Active targeting is based on the specific recognition between receptor and ligand or the covalent modification of targeting groups on the surface. Mackiewicz et al. (55) designed multifunctional poly(ethylene glycol)-block-poly(lactic acid)) nanoparticles modified by folic acid and fluorescent probes, which can achieve the purposes of cell imaging and targeted delivery of anti-tumor drugs at the same time.

### Physical and Chemical Targeted Transport

The microenvironment of tumor cells is different from that of normal cells. Based on the unique physical and chemical environment of tumor site, researchers have developed a series of nano-drug carriers with stimulus response, which can achieve targeted release by controlling exogenous stimulus (change of temperature, magnetic field, light or electric pulse) or endogenous stimulus (change of PH value or redox), thereby improving drug efficacy and reducing side effects (56–59).

#### Magnetic-Responsive Nanocarriers

Since Widder et al. (60) proposed the targeted therapy of magnetic drugs in the 1970s, the research on magnetic targeted drug delivery system (MTDS) has become an important part of the current research on tumor diagnosis and treatment. Magnetic nanoparticles were fixed by external magnetic field, and then heated by alternating magnetic field to kill tumor cells (61). MTDS usually used core-shell nanoparticles (62), magnetic liposomes (63) and nanoporous metal capsules (64) as magnetic responsive nanocarriers. Among them, superparamagnetic iron oxide nano drug-loaded particles have become the current research focus due to their low cytotoxicity, chemical and magnetic stability. Shalaby et al. (65) combined magnetic nanoparticles with adenovirus to transfect them into human fibroma cells under the action of external magnetic field. The results showed that magnetic transfection could significantly inhibit cell proliferation and induce apoptosis.

#### PH-Responsive Nanocarriers

pH value in tumor cells is generally lower than that in normal tissues. The pH value of normal tissue is about 7.4, while the pH value of tumor extracellular microenvironment is about 6.5~7.2 (66). Researchers have developed pH-responsive drug carrier systems by introducing alkaline polymers containing multiple amino groups into polymers, or acetals, orthoesters, hydrazine bonds that can be broken in acidic environments (67–69). This carrier systems were often used to control drug release in specific organs (such as the gastrointestinal tract or vagina) or organelles (such as nucleosomes or lysosomes) and trigger drug release when microenvironment changes are associated with pathological conditions (70). Deng et al. (71) found that the amino protonation caused by chitosan swelling would lead to the release of tumor necrosis factor-α (TNF-α) encapsulated in chitosan in tumor tissues in local acidic environment.

#### Temperature-Responsive Nanocarriers

Temperature-responsive nanocarriers maintain structural integrity in normal tissues (37°C), and the drug is well encapsulated in nanomaterials, but the tumor tissue temperature of the patients treated by hyperthermia therapy is as high as 39.5°C, therefore, when the nano-carrier reaches the tumor site, at least one component of the nano-material responds to the nonlinear rapid change of temperature, the structure of the system is destroyed and the drug is released, thus realizing targeted drug delivery at the tumor site (72). Temperature-responsive nanocarriers usually included liposomes and polymer micelles (n-isopropylacrylamide). Shah et al. (73) wrapped photosensitizer tetrakis(hydroxymethyl)phosphonium chloride and anticancer drug doxorubicin in hydrophobic lipid bilayer membrane, and wrapped magnetic nanoparticles in hydrophilic inner capsule, realizing simultaneous magnetocaloric therapy, photodynamic therapy and chemotherapy. Experimental results show that combined therapy can almost eliminate completely cancer cells, and the therapeutic effect is remarkable.

#### Photo-Responsive Nano-Carrier

The photo-responsive nano-carrier can respond to specific wavelength light to achieve targeted drug delivery (74). The photosensitive azobenzene group and its derivatives can be optically isomerized from trans to cis under the irradiation of 300-380 nm, and can also be optically isomerized from cis to trans in the visible region, which makes it possible to optically control drug release (75). Because soft tissue has strong scattering in the ultraviolet visible region less than 700 nm, the penetration depth of light-responsive nanocarriers is low (~ 10 mm), which is only suitable for body parts that can be directly irradiated (such as eyes and skin, etc.) (74). Today, near infrared lasers (NIR) with wavelengths ranging from 700 to 1000 nm have wide clinical applications due to their low scattering, deep tissue penetration, and micro-tissue damage (76). YOU et al. (77) designed and synthesized multifunctional doxorubicin hollow gold nanoparticles, which accelerated the release of drugs under the irradiation of near infrared light. Compared with traditional chemotherapy methods, the anti-cancer activity was increased, and the systemic toxicity was reduced, proving that the NIR technology has a broad prospect.

#### Redox-Responsive Nanocarriers

Concentration of Glutathione (GSH) in tumor cells was about several hundred times higher than that in extracellular cells. In general, redox-responsive nanocarriers can intelligently target drug release in tumor cells by introducing disulfide or diselenide bonds (78), which is of great significance to many drug molecules (e. g. camptothecin, doxorubicin, etc.) that exert their effects in the organelles of tumor cells. Disulfide bonds are very stable under normal physiological condition but will be reduced and broken in the presence of high concentration GSH in tumor cells (79). Based on this principle, Wang et al. (80) developed Camptothecin (CPT) conjugated core cross-linked micelles that can break down disulfide bonds by oxidation-reduction, thus, destroying the micelle structure and releasing CPT rapidly. In vitro cytotoxicity study showed that the anti-cancer activity of redox-responsive core cross-linked micelles was significantly higher than that of nonresponsive micelles.

## Conclusion and Prospect

Great progress has been made in the research of nano anti-tumor drug carrier, which effectively overcomes the limitations of poor solubility, low bioavailability and non-specificity of traditional chemotherapy drugs, and obviously improves the curative effect of drugs. At present, nano-carriers for anti-tumor drugs mainly include nano-liposomes, nano-polymers and nano-gene carriers, which belong to nano-organic materials and nano-inorganic materials. The application of anti-tumor drug nanocarriers can truly achieve targeted drug delivery at the focus, and its targeted transportation modes mainly include passive transportation, active transportation and physical and chemical transportation (according to the changes of temperature, magnetic field, light or electric pulse, PH and redox). However, most of the research on nano anti-tumor drug carrier focuses on the basic theory, and few drugs can be used clinically, which limits the wide application of nano anti-tumor drug carriers (81–83). In the future, researchers need to continuously explore and design nanoporous carriers of anti-tumor drugs with high drug loading, high efficiency, low toxicity, low cost and clinical application value. The modification of nanoparticles is combined with single surface group modification and combined physicochemical modification so as to establish a new route of administration, which is conducive to the interdisciplinary comprehensive research and maximize its scientific value and market value.

## Author Contributions

JX wrote the paper. ZR participated in the design and drafting of this manuscript. BW, XS and XG performed the literature search and revised the paper. WL and ST guided the writing of the paper and reviewed the manuscript. All authors contributed to the article and approved the submitted version.

## Conflict of Interest

The authors declare that the research was conducted in the absence of any commercial or financial relationships that could be construed as a potential conflict of interest.

The handling editor declared a shared affiliation, though no other collaboration with the authors at the time of the review.

## Publisher’s Note

All claims expressed in this article are solely those of the authors and do not necessarily represent those of their affiliated organizations, or those of the publisher, the editors and the reviewers. Any product that may be evaluated in this article, or claim that may be made by its manufacturer, is not guaranteed or endorsed by the publisher.
